# *catena*-Poly[[bis­(quinolin-8-amine-κ^2^ *N*,*N*′)cadmium(II)]-μ-cyanido-κ^2^ *N*:*C*-[dicyanidonickel(II)]-μ-cyanido-κ^2^ *C*:*N*]

**DOI:** 10.1107/S241431462100568X

**Published:** 2021-06-08

**Authors:** Selma Khelfa, Marwa Touil, Zouaoui Setifi, Fatima Setifi, Mohammed Hadi Al-Douh, Christopher Glidewell

**Affiliations:** a Département de Chimie, Faculté des Sciences, Université 20 Août 1955-Skikda, BP 26, Route d’El-Hadaiek, Skikda 21000, Algeria; b Laboratoire de Chimie, Ingénierie Moléculaire et Nanostructures (LCIMN), Université Ferhat Abbas Sétif 1, Sétif 19000, Algeria; c Département de Technologie, Faculté de Technologie, Université 20 Août 1955-Skikda, BP 26, Route d’El-Hadaiek, Skikda 21000, Algeria; d Chemistry Department, Faculty of Science, Hadhramout University, Mukalla, Hadhramout, Yemen; e School of Chemistry, University of St Andrews, St Andrews, Fife KY16 9ST, UK; Goethe-Universität Frankfurt, Germany

**Keywords:** synthesis, crystal structure, coordination polymer, hydrogen bonding

## Abstract

In the title compound, [CdNi(C_9_H_8_N_2_)_2_(CN)_4_]_*n*_, the Cd and Ni atoms both lie on centres of inversion in space group *P*2_1_/*c*. The Cd atom is coordinated by two bidentate quinolin-8-amine ligands and by the N atoms of two cyano ligands, while the square planar Ni atom is coordinated by the C atoms of four cyano ligands. These units form a one-dimensional coordination polymer containing an (–NC—Ni—CN—Cd–)_*n*_ backbone, and the coordination polymer chains are linked into a three-dimensional array by a combination of N—H⋯N and C—H⋯N hydrogen bonds, augmented by a π–π stacking inter­action.

## Abstract 2

The title compound forms a one-dimensional coordination polymer containing alternating six-coordinate Cd and four-coordinate Ni atoms, linked by bridging cyano ligands

## Graphical abstract

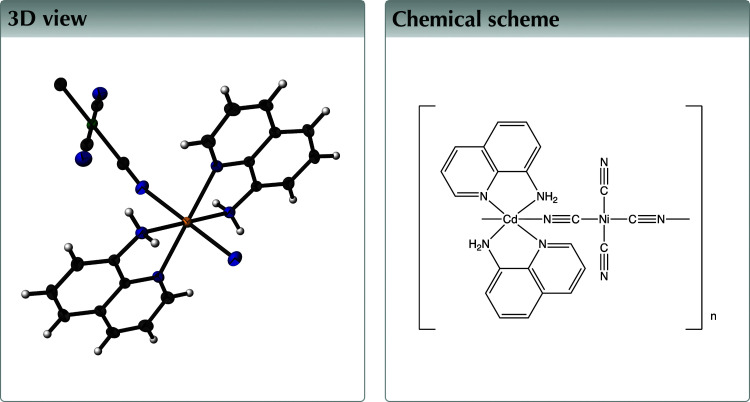

## Structure description

Transition-metal coordination compounds in which cyano ligands play the main structure-forming role, so-called cyano­carbanion or cyano­metallate complexes, have been the subject of inter­est for many years, because of their magnetic and luminescent properties (Sieklucka *et al.*, 2011 ▸; Benmansour *et al.*, 2007 ▸, 2008 ▸, 2009 ▸, 2012 ▸; Setifi *et al.*, 2009 ▸; Yuste *et al.*, 2009 ▸; Lehchili *et al.*, 2017 ▸) including, in particular, their spin-crossover behaviour (Benmansour *et al.*, 2010 ▸; Setifi *et al.*, 2013 ▸, 2014 ▸, Bartual-Murgui *et al.*, 2013 ▸). In a continuation of our general study of this area, we now report the crystal and mol­ecular structure of the title compound.

**Figure 1:**
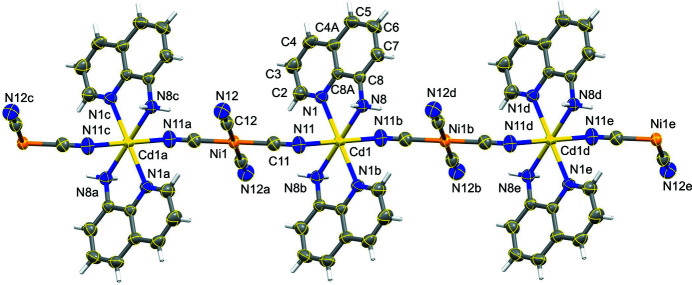
The coordination polymer formed by the title compound. For the sake of clarity many of the C atom labels have been omitted: the atoms marked with a, b, c, d or e are at the symmetry positions (1 − *x*, 1 − *y*, −*z*), (1 − *x*, 1 − *y*, 1 − *z*), (*x*, *y*, −1 + *z*), (*x*, *y*, 1 + *z*) and (1 − *x*, 1 − *y*, 2 − *z*), respectively,. Displacement ellipsoids are drawn at the 80% probability level.

**Figure 2:**
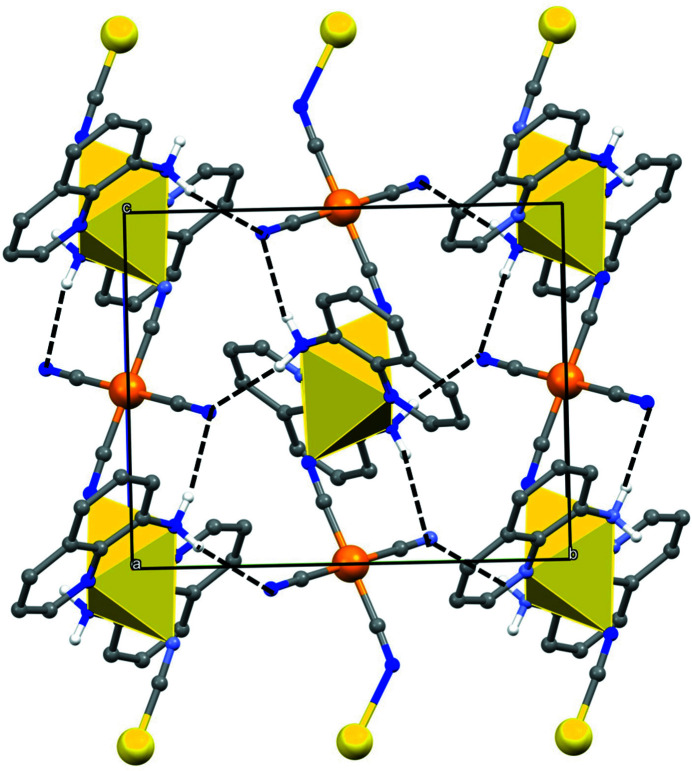
A projection along [100] of part of the crystal structure showing the formation of a hydrogen-bonded sheet of polymer chains, lying parallel to (100). For the sake of clarity, the H atoms bonded to C atoms have been omitted.

**Figure 3:**
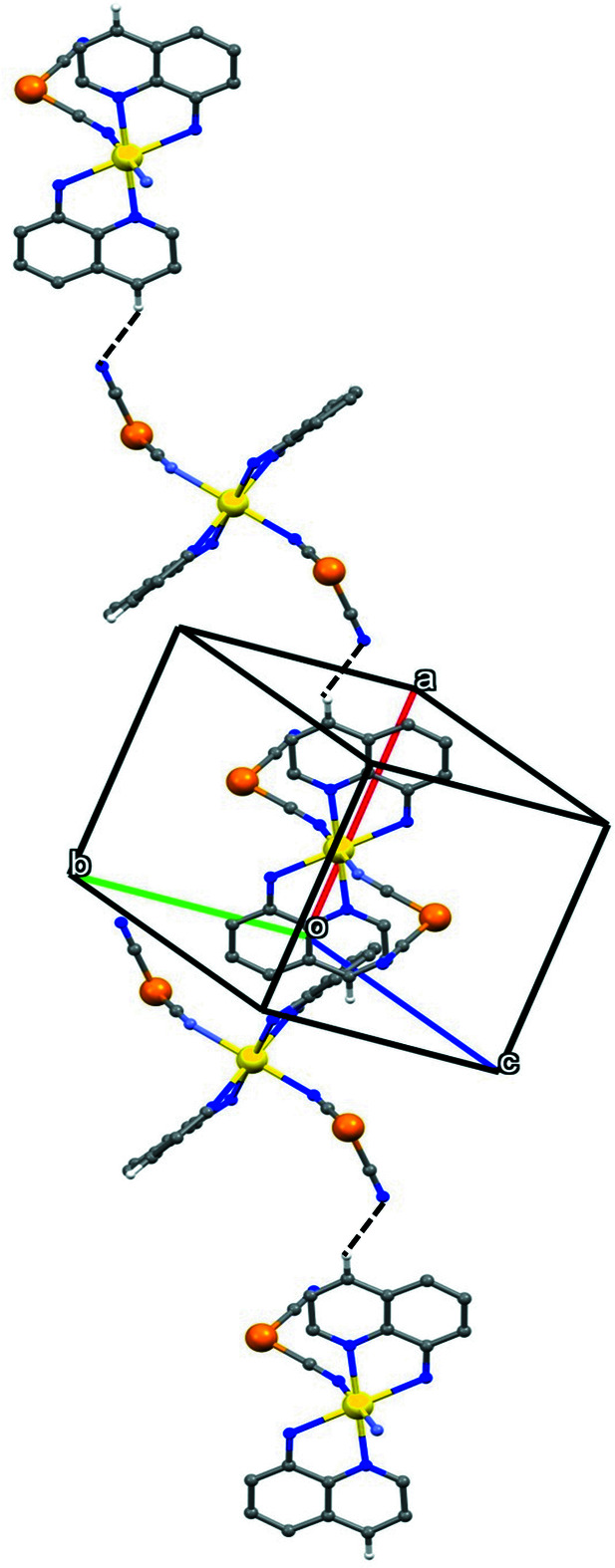
Part of the crystal structure showing the formation of a hydrogen-bonded chain running parallel to the [20 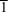 ] direction. For the sake of clarity, the H atoms not involved in the motif shown have been omitted.

**Figure 4:**
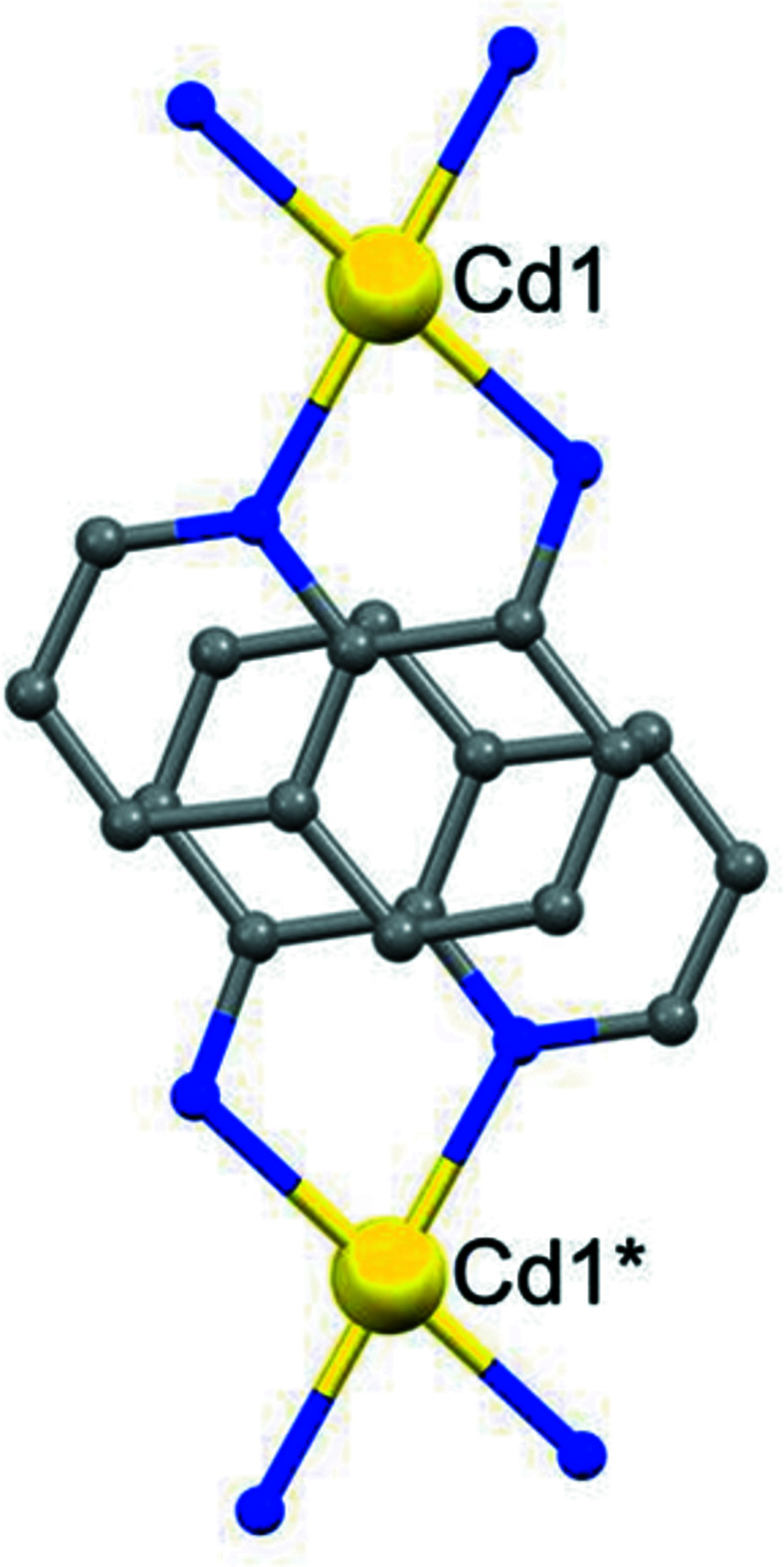
Part of the crystal structure showing the π-stacking of quinolin-8-amine ligands. For the sake of clarity, the unit-cell outline and the H atoms have been omitted: the Cd atom marked with an asterisk (*) is at (1.5, 1/2, 1/2).

In the structure of the title compound, the Cd and Ni ions both lie on centres of inversion, selected for convenience as those at (0.5, 0.5, 0.5) and (0.5, 0.5, 0), respectively. The [Ni(CN)_4_]^2−^ units adopts the usual square planar configuration, while the Cd centre is coordinated by two bidentate quinolin-8-amine units and by the N atoms of two cyano ligands. The structure thus consists of one-dimensional coordination polymer based on an (–NC—Ni—CN—Cd–)_*n*_ backbone and running parallel to [001]. In the reference chain [Cd{quinolin-8-amine)_2_]^2+^ units centred at (0.5, 0.5, *n* + 0.5) alternate with [Ni(CN)_4_]^2−^ units centred at (0.5, 0.5, *n*), where *n* represents an integer in each case (Fig. 1 ▸). There are two types of N—H⋯N hydrogen bond in the structure (Table 1 ▸). Those involving atom H8*A* lie within the coordination polymer chain, but those involving atom H8*B* link the chain along (0.5, 0.5, *z*) to those along (0.5, 0, z) and (0.5, 1, *z*), so forming a sheet of hydrogen-bonded chains lying parallel to (100) (Fig. 2 ▸). Sheets of this type are linked into a three-dimensional array by two types of direction-specific inter­actions, a C—H⋯N hydrogen bond (Table 1 ▸) and a π–π stacking inter­action. The C—H⋯N hydrogen bond combines with the inversion symmetry at both metal centres to generate a chain running parallel to the [20 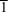 ] direction (Fig. 3 ▸), which links the (100) sheets into a three-dimensional structure. In addition, the carbocyclic rings in the quinolin-8-amine ligands at (*x*, *y*, *z*) and (2 − *x*, 1 − *y*, 1 − *z*), which lie in adjacent (100) sheets, are strictly parallel with an inter­planar spacing of 3.4070 (6) Å; the ring-centroid separation is 3.5856 (8) Å, with a ring-centroid offset of *ca* 1.117 (2) Å: the inter­actions between the two types of ring in these two ligands are similar (Fig. 4 ▸).

The structure of the title compound is very similar to that of the iron(II)–nickel analogue, whose structure has been studied at both 293 K and 120 K, where the iron adopts high-spin and low-spin configurations, respectively (Setifi *et al.*, 2014 ▸). This structural similarity of the Cd^II^ and Fe^II^ compounds is somewhat unexpected in view of the different effective radii of these ions (Shannon & Prewitt, 1969 ▸, 1970 ▸), reflected in the differences between the *M*—N (*M* = Cd or Fe) distances in the two compounds, typically around 0.30 Å for each type of bond, itself reflected in the difference between the a repeat vectors, 9.4264 (3) Å for *M* = Cd but only 9.0035 (5) Å for *M* = Fe at 120 K.

## Synthesis and crystallization

A solution of quinolin-8-amine (0.288 g, 2 mmol) in ethanol (10 ml) was added dropwise with stirring at 323 K to a solution of Cd[Ni(CN)_4_]·H_2_O (0.293 g, 1 mmol) in water (10 ml). This mixture was stirred for 4 h at 323 K and then filtered. Slow evaporation of the filtrate over a period of one week, at ambient temperature and in the presence of air, gave crystals suitable for single-crystal X-ray diffraction.

## Refinement

Crystal data, data collection and structure refinement details are summarized in Table 2 ▸.

## Supplementary Material

Crystal structure: contains datablock(s) global, I. DOI: 10.1107/S241431462100568X/bt4114sup1.cif

Structure factors: contains datablock(s) I. DOI: 10.1107/S241431462100568X/bt4114Isup2.hkl

CCDC reference: 2087407

Additional supporting information:  crystallographic information; 3D view; checkCIF report

## Figures and Tables

**Table 1:** Hydrogen-bond geometry (Å, °) *Cg*1 is the centroid of the C4*A*/C5–C8/C8*A* ring.

*D*—H⋯*A*	*D*—H	H⋯*A*	*D*⋯*A*	*D*—H⋯*A*
N8—H8*A*⋯N12^i^	0.880 (18)	2.416 (18)	3.2815 (18)	167.8 (15)
N8—H8*B*⋯N12^ii^	0.867 (19)	2.286 (19)	3.1275 (17)	163.9 (17)
C3—H3⋯*Cg*1^iii^	0.95	2.78	3.6266 (17)	149
C4—H4⋯N12^iv^	0.95	2.58	3.443 (2)	151

Symmetry codes: (i) 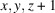 ; (ii) 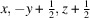 ; (iii) 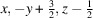 ; (iv) 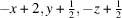 .

**Table 2:** Experimental details

Crystal data	
Chemical formula	[CdNi(C_9_H_8_N_2_)_2_(CN)_4_]
*M*_r_	563.53
Crystal system, space group	Monoclinic, *P*2_1_/*c*
Temperature (K)	170
*a*, *b*, *c* (Å)	9.4264 (3), 11.8622 (3), 9.8257 (3)
β (°)	101.088 (2)
*V* (Å^3^)	1078.18 (6)
*Z*	2
Radiation type	Mo *K*α
μ (mm^−1^)	1.89
Crystal size (mm)	0.15 × 0.11 × 0.07

Data collection	
Diffractometer	Rigaku Oxford Diffraction Xcalibur, Eos, Gemini
Absorption correction	Multi-scan (*CrysAlis PRO*; Rigaku OD, 2015 ▸)
*T*_min_, *T*_max_	0.668, 0.885
No. of measured, independent and observed [*I* > 2σ(*I*)] reflections	22794, 4057, 3319
*R*_int_	0.024
(sin θ/λ)_max_ (Å^−1^)	0.770

Refinement	
*R*[*F* ^2^ > 2σ(*F* ^2^)], *wR*(*F* ^2^), *S*	0.022, 0.063, 1.07
No. of reflections	4057
No. of parameters	154
H-atom treatment	H atoms treated by a mixture of independent and constrained refinement
Δρ_max_, Δρ_min_ (e Å^−3^)	0.55, −0.51

Computer programs: *CrysAlis PRO* (Rigaku OD, 2015 ▸), *SHELXS86* (Sheldrick, 2015 ▸), *SHELXL2014* (Sheldrick, 2015 ▸) and *PLATON* (Spek, 2020 ▸).

## full crystallographic data

### Crystal data

**Table:**

[CdNi(C_9_H_8_N_2_)_2_(CN)_4_]	*F*(000) = 560
*M**_r_* = 563.53	*D*_x_ = 1.736 Mg m^−^^3^
Monoclinic, *P*2_1_/*c*	Mo *K*α radiation, λ = 0.71073 Å
*a* = 9.4264 (3) Å	Cell parameters from 4057 reflections
*b* = 11.8622 (3) Å	θ = 2.8–33.2°
*c* = 9.8257 (3) Å	µ = 1.89 mm^−^^1^
β = 101.088 (2)°	*T* = 170 K
*V* = 1078.18 (6) Å^3^	Block, pale yellow
*Z* = 2	0.15 × 0.11 × 0.07 mm

### Data collection

**Table:**

Rigaku Oxford Diffraction Xcalibur, Eos, Gemini diffractometer	4057 independent reflections
Radiation source: fine-focus sealed X-raytube	3319 reflections with *I* > 2σ(*I*)
Graphite monochromator	*R*_int_ = 0.024
ω scans	θ_max_ = 33.2°, θ_min_ = 2.8°
Absorption correction: multi-scan (CrysAlis PRO; Rigaku OD, 2015)	*h* = −14→12
*T*_min_ = 0.668, *T*_max_ = 0.885	*k* = −18→18
22794 measured reflections	*l* = −12→15

### Refinement

**Table:**

Refinement on *F*^2^	Primary atom site location: difference Fourier map
Least-squares matrix: full	Hydrogen site location: mixed
*R*[*F*^2^ > 2σ(*F*^2^)] = 0.022	H atoms treated by a mixture of independent and constrained refinement
*wR*(*F*^2^) = 0.063	*w* = 1/[σ^2^(*F*_o_^2^) + (0.0239*P*)^2^ + 0.6589*P*] where *P* = (*F*_o_^2^ + 2*F*_c_^2^)/3
*S* = 1.07	(Δ/σ)_max_ < 0.001
4057 reflections	Δρ_max_ = 0.55 e Å^−^^3^
154 parameters	Δρ_min_ = −0.50 e Å^−^^3^
0 restraints	

### Special details

**Table:**

Geometry. All esds (except the esd in the dihedral angle between two l.s. planes) are estimated using the full covariance matrix. The cell esds are taken into account individually in the estimation of esds in distances, angles and torsion angles; correlations between esds in cell parameters are only used when they are defined by crystal symmetry. An approximate (isotropic) treatment of cell esds is used for estimating esds involving l.s. planes.

### Fractional atomic coordinates and isotropic or equivalent isotropic displacement parameters (Å^2^)

**Table:**

	*x*	*y*	*z*	*U*_iso_*/*U*_eq_	
Cd1	0.5000	0.5000	0.5000	0.01846 (4)	
N1	0.69972 (12)	0.60058 (9)	0.46816 (12)	0.0200 (2)	
C2	0.69877 (16)	0.69047 (12)	0.38871 (16)	0.0259 (3)	
H2	0.6118	0.7094	0.3266	0.031*	
C3	0.82124 (18)	0.75965 (12)	0.39193 (18)	0.0286 (3)	
H3	0.8173	0.8224	0.3312	0.034*	
C4	0.94535 (17)	0.73487 (12)	0.48371 (16)	0.0246 (3)	
H4	1.0286	0.7809	0.4878	0.029*	
C4A	0.94994 (14)	0.64063 (11)	0.57272 (13)	0.0200 (2)	
C5	1.07330 (15)	0.61251 (13)	0.67437 (15)	0.0252 (3)	
H5	1.1583	0.6572	0.6842	0.030*	
C6	1.06942 (16)	0.52108 (14)	0.75798 (16)	0.0275 (3)	
H6	1.1505	0.5045	0.8288	0.033*	
C7	0.94610 (15)	0.45098 (13)	0.74017 (14)	0.0239 (3)	
H7	0.9465	0.3868	0.7981	0.029*	
C8	0.82593 (14)	0.47349 (11)	0.64121 (13)	0.0181 (2)	
C8A	0.82395 (13)	0.57249 (10)	0.55881 (12)	0.0172 (2)	
N8	0.70170 (12)	0.40018 (9)	0.61705 (12)	0.0198 (2)	
H8A	0.694 (2)	0.3681 (15)	0.6961 (19)	0.024*	
H8B	0.713 (2)	0.3478 (16)	0.5586 (19)	0.024*	
Ni1	0.5000	0.5000	0.0000	0.01665 (5)	
C11	0.50450 (15)	0.44216 (11)	0.17628 (14)	0.0217 (2)	
N11	0.50472 (15)	0.41011 (11)	0.28716 (13)	0.0272 (2)	
C12	0.62164 (15)	0.38568 (12)	−0.03912 (14)	0.0219 (2)	
N12	0.69773 (15)	0.31621 (11)	−0.06549 (14)	0.0292 (3)	

### Atomic displacement parameters (Å^2^)

**Table:**

	*U*^11^	*U*^22^	*U*^33^	*U*^12^	*U*^13^	*U*^23^
Cd1	0.01554 (6)	0.02316 (7)	0.01642 (7)	−0.00368 (4)	0.00242 (4)	0.00056 (4)
N1	0.0186 (5)	0.0198 (5)	0.0214 (5)	−0.0013 (4)	0.0032 (4)	0.0026 (4)
C2	0.0242 (6)	0.0248 (6)	0.0282 (7)	−0.0007 (5)	0.0040 (5)	0.0077 (5)
C3	0.0302 (7)	0.0236 (6)	0.0329 (8)	−0.0038 (5)	0.0088 (6)	0.0078 (5)
C4	0.0241 (7)	0.0227 (6)	0.0287 (7)	−0.0071 (5)	0.0098 (5)	−0.0012 (5)
C4A	0.0184 (5)	0.0209 (5)	0.0214 (5)	−0.0029 (4)	0.0059 (4)	−0.0036 (5)
C5	0.0182 (6)	0.0307 (7)	0.0264 (6)	−0.0041 (5)	0.0032 (5)	−0.0058 (5)
C6	0.0196 (6)	0.0353 (7)	0.0250 (7)	−0.0001 (5)	−0.0023 (5)	−0.0013 (6)
C7	0.0240 (6)	0.0249 (6)	0.0217 (6)	0.0015 (5)	0.0015 (5)	0.0025 (5)
C8	0.0186 (5)	0.0188 (5)	0.0172 (5)	−0.0005 (4)	0.0044 (4)	−0.0011 (4)
C8A	0.0175 (5)	0.0175 (5)	0.0172 (5)	−0.0010 (4)	0.0045 (4)	−0.0012 (4)
N8	0.0223 (5)	0.0171 (5)	0.0202 (5)	−0.0020 (4)	0.0045 (4)	0.0005 (4)
Ni1	0.01888 (11)	0.01653 (10)	0.01467 (10)	0.00374 (8)	0.00356 (8)	−0.00017 (7)
C11	0.0235 (6)	0.0203 (5)	0.0213 (6)	0.0032 (5)	0.0041 (5)	−0.0022 (5)
N11	0.0343 (7)	0.0269 (6)	0.0205 (5)	0.0014 (5)	0.0056 (4)	−0.0007 (5)
C12	0.0244 (6)	0.0217 (6)	0.0196 (5)	0.0029 (5)	0.0040 (4)	0.0016 (5)
N12	0.0300 (6)	0.0264 (6)	0.0326 (6)	0.0073 (5)	0.0094 (5)	0.0018 (5)

### Geometric parameters (Å, º)

**Table:**

Cd1—N1	2.3005 (11)	C5—C6	1.365 (2)
Cd1—N1^i^	2.3005 (11)	C5—H5	0.9500
Cd1—N8	2.3449 (12)	C6—C7	1.412 (2)
Cd1—N8^i^	2.3449 (12)	C6—H6	0.9500
Cd1—N11^i^	2.3554 (13)	C7—C8	1.3698 (19)
Cd1—N11	2.3555 (13)	C7—H7	0.9500
N1—C2	1.3205 (17)	C8—C8A	1.4245 (18)
N1—C8A	1.3691 (17)	C8—N8	1.4412 (17)
C2—C3	1.412 (2)	N8—H8A	0.880 (19)
C2—H2	0.9500	N8—H8B	0.868 (19)
C3—C4	1.365 (2)	Ni1—C11	1.8559 (14)
C3—H3	0.9500	Ni1—C11^ii^	1.8560 (14)
C4—C4A	1.4149 (19)	Ni1—C12	1.8631 (13)
C4—H4	0.9500	Ni1—C12^ii^	1.8631 (13)
C4A—C5	1.4192 (19)	C11—N11	1.1536 (18)
C4A—C8A	1.4212 (17)	C12—N12	1.1542 (18)
			
N1—Cd1—N1^i^	180.0	C6—C5—C4A	119.80 (13)
N1—Cd1—N8	73.80 (4)	C6—C5—H5	120.1
N1^i^—Cd1—N8	106.20 (4)	C4A—C5—H5	120.1
N1—Cd1—N8^i^	106.20 (4)	C5—C6—C7	120.64 (14)
N1^i^—Cd1—N8^i^	73.80 (4)	C5—C6—H6	119.7
N8—Cd1—N8^i^	180.0	C7—C6—H6	119.7
N1—Cd1—N11^i^	92.38 (4)	C8—C7—C6	121.45 (14)
N1^i^—Cd1—N11^i^	87.62 (4)	C8—C7—H7	119.3
N8—Cd1—N11^i^	86.85 (4)	C6—C7—H7	119.3
N8^i^—Cd1—N11^i^	93.15 (4)	C7—C8—C8A	118.87 (12)
N1—Cd1—N11	87.62 (4)	C7—C8—N8	122.28 (12)
N1^i^—Cd1—N11	92.38 (4)	C8A—C8—N8	118.85 (11)
N8—Cd1—N11	93.15 (4)	N1—C8A—C4A	121.24 (11)
N8^i^—Cd1—N11	86.85 (4)	N1—C8A—C8	119.10 (11)
N11^i^—Cd1—N11	180.0	C4A—C8A—C8	119.66 (12)
C2—N1—C8A	119.26 (12)	C8—N8—Cd1	109.42 (8)
C2—N1—Cd1	125.91 (9)	C8—N8—H8A	108.3 (12)
C8A—N1—Cd1	113.91 (8)	Cd1—N8—H8A	117.2 (12)
N1—C2—C3	122.93 (14)	C8—N8—H8B	109.8 (12)
N1—C2—H2	118.5	Cd1—N8—H8B	103.4 (12)
C3—C2—H2	118.5	H8A—N8—H8B	108.4 (17)
C4—C3—C2	118.87 (13)	C11—Ni1—C11^ii^	180.0
C4—C3—H3	120.6	C11—Ni1—C12	91.08 (6)
C2—C3—H3	120.6	C11^ii^—Ni1—C12	88.92 (6)
C3—C4—C4A	119.94 (13)	C11—Ni1—C12^ii^	88.92 (6)
C3—C4—H4	120.0	C11^ii^—Ni1—C12^ii^	91.08 (6)
C4A—C4—H4	120.0	C12—Ni1—C12^ii^	180.0
C4—C4A—C5	122.97 (13)	N11—C11—Ni1	177.27 (13)
C4—C4A—C8A	117.66 (12)	C11—N11—Cd1	133.82 (11)
C5—C4A—C8A	119.37 (12)	N12—C12—Ni1	178.59 (13)
			
C8A—N1—C2—C3	0.4 (2)	Cd1—N1—C8A—C4A	−167.20 (9)
Cd1—N1—C2—C3	168.70 (12)	C2—N1—C8A—C8	−178.04 (12)
N1—C2—C3—C4	−1.9 (2)	Cd1—N1—C8A—C8	12.30 (14)
C2—C3—C4—C4A	0.5 (2)	C4—C4A—C8A—N1	−3.70 (18)
C3—C4—C4A—C5	−177.41 (14)	C5—C4A—C8A—N1	175.86 (12)
C3—C4—C4A—C8A	2.1 (2)	C4—C4A—C8A—C8	176.80 (12)
C4—C4A—C5—C6	179.15 (14)	C5—C4A—C8A—C8	−3.63 (18)
C8A—C4A—C5—C6	−0.4 (2)	C7—C8—C8A—N1	−174.40 (12)
C4A—C5—C6—C7	2.9 (2)	N8—C8—C8A—N1	5.93 (17)
C5—C6—C7—C8	−1.4 (2)	C7—C8—C8A—C4A	5.11 (18)
C6—C7—C8—C8A	−2.6 (2)	N8—C8—C8A—C4A	−174.57 (11)
C6—C7—C8—N8	177.03 (13)	C7—C8—N8—Cd1	160.34 (11)
C2—N1—C8A—C4A	2.46 (19)	C8A—C8—N8—Cd1	−20.00 (13)

Symmetry codes: (i) −*x*+1, −*y*+1, −*z*+1; (ii) −*x*+1, −*y*+1, −*z*.

### Hydrogen-bond geometry (Å, º)

*Cg*1 is the centroid of the C4*A*/C5–C8/C8*A* ring.

**Table:**

*D*—H···*A*	*D*—H	H···*A*	*D*···*A*	*D*—H···*A*
N8—H8*A*···N12^iii^	0.880 (18)	2.416 (18)	3.2815 (18)	167.8 (15)
N8—H8*B*···N12^iv^	0.867 (19)	2.286 (19)	3.1275 (17)	163.9 (17)
C3—H3···*Cg*1^v^	0.95	2.78	3.6266 (17)	149
C4—H4···N12^vi^	0.95	2.58	3.443 (2)	151

Symmetry codes: (iii) *x*, *y*, *z*+1; (iv) *x*, −*y*+1/2, *z*+1/2; (v) *x*, −*y*+3/2, *z*−1/2; (vi) −*x*+2, *y*+1/2, −*z*+1/2.
